# Comparative Effects of Direct Renin Inhibitor and Angiotensin Receptor Blocker on Albuminuria in Hypertensive Patients with Type 2 Diabetes. A Randomized Controlled Trial

**DOI:** 10.1371/journal.pone.0164936

**Published:** 2016-12-29

**Authors:** Takashi Uzu, Shin-ichi Araki, Atsunori Kashiwagi, Masakazu Haneda, Daisuke Koya, Hiroki Yokoyama, Yasuo Kida, Motoyoshi Ikebuchi, Takaaki Nakamura, Masataka Nishimura, Noriko Takahara, Toshiyuki Obata, Nobuyuki Omichi, Katsuhiko Sakamoto, Ryosuke Shingu, Hideki Taki, Yoshio Nagai, Hiroaki Tokuda, Munehiro Kitada, Miwa Misawa, Akira Nishiyama, Hiroyuki Kobori, Hiroshi Maegawa, The Shiga Committee for Preventing Diabetic Nephropathy

**Affiliations:** 1 Division of Nephrology & Blood Purification, Nissay Hospital, Osaka, Japan; 2 Department of Medicine, Sihiga Univ. of Medical Science, Otsu, Shiga, Japan; 3 Department of Medicine, Kusatsu General Hospital, Kusatsu, Shiga, Japan; 4 Department of Medicine, Asahikawa Medical Univ., Asahikawa, Hokkaido, Japan; 5 Department of Diabetology and Endocrinology, Kanazawa Medical Univ., Kahoku-gun, Ishikawa, Japan; 6 Department of Internal Medicine, Jiyugaoka Medical Clinic, Obihiro, Hokkaido, Japan; 7 Department of Medicine, Daini-Okamoto Hospital, Uji, Kyoto, Japan; 8 Department of Internal Medicine, Ikebuchi Clonic, Osaka, Japan; 9 Department of Endicrinology and Metabolism, Omi Hachiman Community Medical Center, Omi Hachiman, Shiga, Japan; 10 Department of Medicine, Nagahama City Hospital, Nagahama, Shiga, Japan; 11 Department of Medicine, Ako City Hospital, Ako, Hyogo, Japan; 12 Department of Medicine, Omichi Clinic, Otsu, Shiga, Japan; 13 Department of Medicine, Sakamoto Clinic, Minoh, Osaka, Iapan; 14 Department of Medicine, Kitahorie Hospital, Osaka, Japan; 15 Diabetes Center, National Hospital Organization National Osaka Hospital, Osaka, Japan; 16 Division of Metabolism and Endocrinology, St. Marianna Univ School of Medicine, Kawasaki, Kanagawa, Japan; 17 Department of Medicine, Tokuda Clinic, Kitamatsu-ura-gun, Nagasaki, Japan; 18 Department of Medicine, Nagahama Red Cross Hospital, Nagahama, Shiga, Japan; 19 Department of Pharmacology, Kagawa Univ. Kida-gun, Kagawa, Japan; 20 Graduate School of Health Science, Kokusai Iryo Fukushi Daigaku, Ohtawara, Tochigi, Japan; Kurume University School of Medicine, JAPAN

## Abstract

**Background:**

In patients with diabetes, albuminuria is a risk marker of end-stage renal disease and cardiovascular events. An increased renin-angiotensin system activity has been reported to play an important role in the pathological processes in these conditions. We compared the effect of aliskiren, a direct renin inhibitor (DRI), with that of angiotensin receptor blockers (ARBs) on albuminuria and urinary excretion of angiotensinogen, a marker of intrarenal renin-angiotensin system activity.

**Methods:**

We randomly assigned 237 type 2 diabetic patients with high-normal albuminuria (10 to <30 mg/g of albumin-to-creatinine ratio) or microalbuminuria (30 to <300 mg/g) to the DRI group or ARB group (any ARB) with a target blood pressure of <130/80 mmHg. The primary endpoint was a reduction in albuminuria.

**Results:**

Twelve patients dropped out during the observation period, and a total of 225 patients were analyzed. During the study period, the systolic and diastolic blood pressures were not different between the groups. The changes in the urinary albumin-to-creatinine ratio from baseline to the end of the treatment period in the DRI and ARB groups were similar (-5.5% and -6.7%, respectively). In contrast, a significant reduction in the urinary excretion of angiotensinogen was observed in the ARB group but not in the DRI group. In the subgroup analysis, a significant reduction in the albuminuria was observed in the ARB group but not in the DRI group among high-normal albuminuria patients.

**Conclusion:**

DRI and ARB reduced albuminuria in hypertensive patients with type 2 diabetes. In addition, ARB, but not DRI, reduced albuminuria even in patients with normal albuminuria. DRI is not superior to ARB in the reduction of urinary excretion of albumin and angiotensinogen.

## Introduction

Diabetic nephropathy is currently the leading cause of end-stage renal disease (ESRD) in the United States [1] and other Western societies [2]. In Japan, diabetic nephropathy became the leading cause of chronic dialysis in 1998, comprising approximately 44% of cases of new patients requiring renal replacement therapy in 2013 [3].

It has been reported that renin-angiotensin system (RAS) activity is elevated both in the circulation and in the renal tissue of patients with diabetic nephropathy [4][5], and increased RAS activity plays an important role in the generation of hypertension and progression of kidney injury [6]. Clinical trial data also shows that the interruption of the RAS with either angiotensin-converting enzyme inhibitor (ACEI) [7] or angiotensin II receptor blocker (ARB) [8][9][10] contributes to reductions in kidney disease events. Therefore, blockade of the generation and action of angiotensin (Ang) II has become the first-line therapy in the management of patients with diabetes mellitus and hypertension [11].

Microalbuminuria in patients with type 2 diabetes is a powerful predictor of ESRD and cardiovascular diseases [11]. Even within the normal range, elevated urinary albumin excretion has been associated with a significantly greater rate of decline in glomerular filtration rate (GFR) than normal excretion [12]. In addition, we [13] and others [14][15] have found that the relief of microalbuminuria in type 2 diabetic patients is an indicator for renal and cardiovascular risk reduction. These findings suggest that albuminuria change is a surrogate marker for clinical outcomes in diabetic nephropathy.

Aliskiren, a long-acting oral direct renin inhibitor (DRI), effectively reduces functional plasma renin activity and blocks the RAS. The drug is approved for the treatment of hypertension. Recent clinical trials have shown that the addition of aliskiren to standard therapy with RAS blockade in patients with type 2 diabetes leads to more adverse events [16][17]. However, there have been no controlled studies comparing the therapeutic effects of aliskiren with ARB for the treatment of diabetes targeting the optimal blood pressure level.

In the present study, we compared the effect of aliskiren with that of ARB on albuminuria in patients with type 2 diabetes. We also investigated the effects of aliskiren and ARB on urinary excretion of angiotensinogen (ATG), which is a marker of intrarenal renin-angiotensin system activity [18].

## Methods

### Participants

We conducted a multi-center, two-arm, randomized, open label, six-month prospective study comparing the effect of aliskiren versus ARB on albuminuria in hypertensive patients with type 2 diabetes in Japan. The participants were between 20 and 75 years of age with hypertension (taking an anti-hypertensive treatment or having a mean sitting systolic blood pressure (BP)/diastolic BP more than 130/80 mmHg), type 2 diabetes, and high-normal albuminuria (HNA) (urinary albumin-to-creatinine ratio [UACR] ≥ 10 and < 30 mg/g) or micro albuminuria (MA) (UACR ≥ 30 and < 300 mg/g). The protocol of this trial and supporting CONSORT checklist are available as supporting information: see S1 Checklist and S1 Protocol.

During the run-in period, we measured the UACR at the first morning spot collections for three consecutive days. MA was defined as more than 2 of these samples falling within the MA range. HNA was defined as the geometric mean of the UACR in the run-in period being within the HNA range for the patients without MA.

The exclusion criteria were severe hypertension (a systolic or diastolic BP of greater than 180 mmHg and 110 mmHg, respectively), malignant hypertension, and known secondary hypertension. Patients with hyperkalemia at baseline (serum potassium > 5.6 mEq/L) or with a history of gastrointestinal tract surgery were also excluded from this study. The study protocol was approved by the ethics committee of Shiga University of Medical Science and was conducted in accordance with the principles of the Declaration of Helsinki. All of the participants provided their written informed consent.

### Procedures

The trial included an eight-week screening period. At the beginning of the screening period, RAS inhibitors (ACEI, ARB, DRI, or aldosterone antagonist) were withdrawn from patients if they had already been administered. Other antihypertensive medications were maintained at the same dosage throughout the study.

The patients with high-normal albuminuria or microalbuminuria were randomly assigned to receive either ARB or aliskiren according to a minimization method by a contract research organization (Kobe CNS, Hyogo Japan). For allocation of the participants, a computer-generated list of random numbers was used. The dose of aliskiren was started at 150 mg/day (once daily); if the BP did not reach the target (systolic BP/diastolic BP < 130/80 mmHg), the dose of aliskiren was doubled. The starting dosages of ARBs were the standard doses in Japan (Table 1); if the BP did not reach the target, the dose was increased (up to the approved highest dosage is Japan). If adequate BP control could not be achieved with the study drugs, another anti-hypertensive drug other than a RAS inhibitor was added, starting at the minimum effective dose.

**Table 1:** Concomitant drugs at baseline.

	DRI	ARB
Anti-hypertensives	58 (52.2)	70 (61.4)
one agent	53 (47.7)	60 (52.6)
two agents	5 (4.5)	10 (8.8)
Calcium channel blocker	56 (50.0)	65 (57.0)
β blocker	4 (3.6)	13 (11.4)
Diuretics	0	1 (0.9)
α blocker	0	1 (0.9)
Insulin	32 (28.8)	25 (21.9)
Sulfonylurea	51 (45.9)	56 (49.1)
DPP-4 inhibitor	49 (44.1)	46 (40.4)
Metformin	39 (35.1)	38 (33.3)
Statin	28 (25.2)	25 (21.2)

The values represent the number of patients (%).

BP was measured using a mercury sphygmomanometer with the patients in the sitting position after at least five minutes of rest. The average of two measurements taken one minute apart was used for analysis. Patients were followed up every 4 weeks for 24 weeks, and blood and urine samples were collected at 12 and 24 weeks. Urine samples were obtained from the first morning void urine sample for three consecutive days. Since the UACR is known to show daily within-person variability, the UACR values were taken as the geometric mean of three consecutive days in each visit in this study. In contrast, the urinary angiotensinogen-to-creatinine ratio (UATGCR) had little day-to-day variability (our preliminary data) and was measured based on one spot urine sample per visit. Urinary albumin, urinary creatinine, serum HbA1c were measured at a central laboratory (SRL Inc., Tokyo, Japan). Urinary angiotensinogen was measured in the Department of Pharmacology at Kagawa University.

### Outcomes

The primary outcome was the percentage change in the geometric mean of the UACR from the baseline. The secondary outcome was the change in the UATGCR from the baseline. Although we also plan to examine the change in the plasma renin activity and serum insulin level, we could not obtain these data during the follow-up period.

### Statistical analysis

We estimated that a sample of 280 participants would give the trial 80% power with 5% significance (2-sided) to detect a reduction of the urinary albumin excretion in the DRI group that was at least 20% lower than that of ARB group using the *t*-test. All of the baseline data are expressed as mean ± standard deviation (SD) or mean (95% confidence interval [CI]). Changes in the ACR and UATGCR were expressed as the median (inter-quartile range). The unpaired Student’s t-test was used to evaluate the differences between two groups. An analysis of variance (ANOVA) was used to evaluate the differences between four groups. Changes in the systolic and diastolic BP were analyzed by ANOVA repeated measures. Changes in the ACR and UATGCR from baseline were analyzed by the Friedman test, and multiple pairwise comparisons were performed by Wilcoxon’s signed‐rank test with Bonferroni correction. A p-value less than 0.05 (two-sided) was deemed to indicate statistical significance. All of the analyses were performed using the IBM SPSS22 Statistics software program. This study is registered with ClinicalTrials.gov, number NCT01461499.

## Results

**Fig 1:**
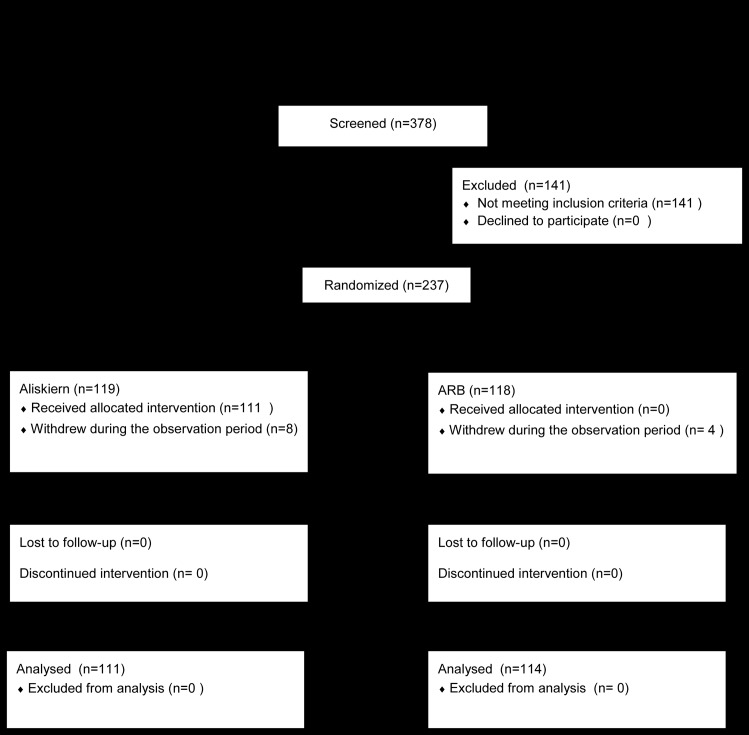
Study subject disposition.

Between December 2011 and November 2013, 378 patients were enrolled, and 237 were randomly assigned to receive either ARB or DRI. Twelve patients (DRI 8, ARB 4) dropped out during the observation period, and a total of 225 patients were studied (Fig 1). The baseline characteristics were similar between the ARB and DRI groups (Table 2). Among the 114 patients assigned to receive ARB, 45 received valsartan (80 mg/day), 26 received telmisartan (40 mg/day), 22 received olmesartan (20 mg/day), 8 received candesartan (8 mg/day), 6 received losartan (50 mg/day), 6 received irbesartan (100 mg/day), and 1 received azilsartan (20 mg/day). Concomitant drugs at baseline are listed in Table 1. Two patients in the ARB group started taking calcium channel blockers during the follow-up period. In the remaining patients, the antihypertensive agents besides DRI or ARB were not changed.

**Table 2:** Baseline characteristics.

	Total		High-normal albuminuria		Microalbuminuria	
	DRI	ARB	DRI(Group 1)	ARB (Group 2)	DRI (Group 3)	ARB (Group 4)
Patients (n)	111	114	62	61	49	53
Age (years)	62.9 ± 9.3	63.0 ± 8.4	61.8 ± 8.5	62.8 ± 8.8	64.3 ± 10.2	63.3 ± 8.0
Female (n [%])	34 (31%)	33 (29%)	21 (34%)	17 (28%)	13 (27%)	16 (30%)
BMI (kg/m^2^)	26.0 ± 4.8	25.4 ± 4.2	26.2 ± 5.0	25.1 ± 4.5	25.7 ± 4.5	25.9 ±4 .2
Systolic BP (mmHg)	137.1 ± 16.5	140.0 ± 14.5	139.2 ± 15.7	138.0 ± 14.0	134.5 ± 17.2	142.4 ± 14.8
Diastolic BP (mmHg)	77.0 ±12.0	76.4 ± 11.2	78.7 ± 11.7	75.9 ± 12.1	74.8 ± 12.1	77.0 ± 10.2
Pulse rate (bpm)	73.6 ± 11.1	75.4 ± 11.8	73.4 ± 12.1	76.0 ± 13.0	73.8 ± 10.0	74.8 ± 10.2
Hb (μmol/L)	1.41 ± 0.14	1.44 ± 0.16	1.42 ± 0.13	1.44 ± 0.17	1.40 ± 0.14	1.42 ± 0.15
TP (g/L)	73 ± 4	73 ± 4	74 ± 4	73 ± 3	72 ± 4	73 ± 4
Tchol (mmpl/L)	5.2 ± 0.9	4.9 ±0.7	5.3 ± 0.9	4.8 ± 0.8	5.0 ± 0.8	5.1 ± 0.5
HDL-C (mmol/L)	1.6 ± 0.5	1.5 ± 0.4	1.6 ± 0.5	1.5 ± 0.5	1.5 ± 0.4	1.6 ± 0.4
Creatinine (μmol/L)	66 ± 16	68 ± 18	65 ± 14	67 ± 17	68 ± 19	72 ± 19
eGFR (ml/min/1.73 m^2^)	79.6 ± 20.7	76.3 ± 21.0	80.0 ± 17.6	78.5 ± 20.0	79.2 ± 24.3	73.9 ± 2.1
Na (mmol/L)	140 ± 2	140 ± 2	140 ± 2	140 ± 2	140 ± 2	140 ± 2
K (nmol/l)	4.3 ± 0.5	4.3 ± 0.4	4.3 ± 0.5	4.2 ± 0.4	4.3 ± 0.4	4.3 ± 0.4
HbA1c (%)	7.0 ± 1.0	6.9 ± 1.0	7.0 ± 1.0	6.9 ± 1.1	7.0 ± 1.0	6.8 ± 0.9
UACR (mg/g)	30.6 (26.1–35.8)	33.0 (27.9–39.0)	16.5 (15.1–18.1)	16.0 (14.8–17.2)	66.6* (56.1–79.2)	76.1* (64.0–90.5)
UATG/Cr (μg/g)	8.15 (5.86–11.3)	15.1* (10.8–21.1)	7.6 (4.7–12.2)	11.6 (7.2–18.8)	8.9 (5.6–14.3)	20.3* (13.1–31.4)

The values represent the mean±standard deviation or mean (95% confidence interval).

* p < 0.05 vs. DRI

† p<0.05 vs. Groups 1 and 2

‡ p< 0.05 vs. Group 1

HNA: high normal albuminuria, MA: microalbuminuria, BP: blood pressure, eGFR: estimated glomerular filtration rate, UACR: urinary albumin-to-creatinine ratio, UATG: urinary angiotensinogen level, Cr: creatinine

**Fig 2:**
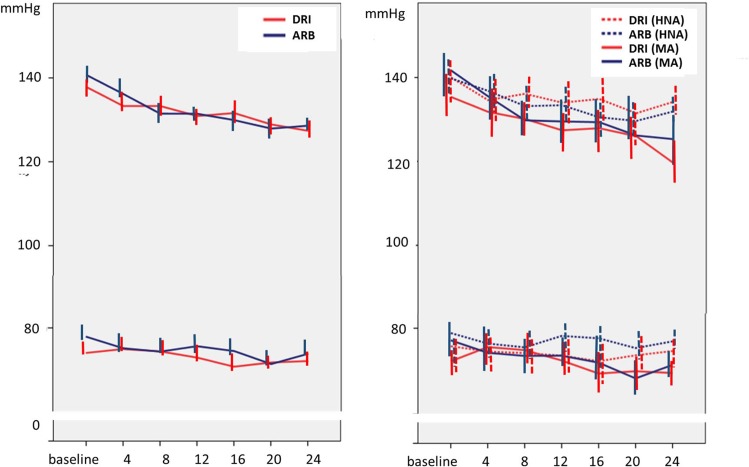
The mean changes in the systolic blood pressure and diastolic blood pressure during follow-up for the two treatment arms (A) and for the subgroups (B). DRI; direct renin inhibitor, ARB; angiotensin receptor blocker, HNA; high normal albuminuria, MA; microalbuminuria. Error bars represent 95% confidential intervals.

During the follow-up period, systolic and diastolic BP were significantly reduced from the baseline period. The mean reductions in trough BP from baseline to Week 24 were similar in both treatment arms (SPB: DRI, −9.3 mm Hg; ARB, −9.4 mm Hg, DBP: −3.9 mmHg, ARB −2.9 mmHg). The time courses of the BP changes were also similar between the two treatment arms for all patients (Fig 2A) as well as subgroups (HNA and MA) (Fig 2B).

The reduction rates of UACR from baseline at Week 24 were 5.5% in the DRI arm and 6.7% in the ARB arm. The treatment effect was not markedly different between the two arms (Table 3). In the ARB arm, significant reductions in the UACR were found in the HNA subgroup and MA subgroup. However, in the subgroup analysis, no significant reductions in the UACR were noted in the DRI arms. The subgroup analysis showed that ARB was beneficial in UACR reduction, but not DRI. A significant reduction in the UATGCR was observed in the ARB arm but not the DRI arm (Table 4). In the ARB arm, significant reductions in the UATGCR were also found in the HNA and MA subgroups.

**Table 3:** Percentage change in the urinary albumin-to-creatinine ratio.

	DRI		ARB	
%Change in UACR (95% CI)	12 weeks	24 weeks	12 weeks	24 weeks
Total	-4.5 (-12.8, 1.7)*	-5.5 (-13.5, 2.5)*	-6.2 (-17.8, 1.7)*	-6.7 (-20.3, 0.6)*
High-normal albuminuria	-3.7 (-7.1, 0.6)	-4.3 (-8.6, 0.2)	-4.1 (-8.8, 1.5)*	-4.8 (-9.6, -0.9)*
Microalbuminuria	-12.0 (-34.2, 18.0)	-11.2 (-29.1, 23.8)	-18.7 (-48.0, 6.1)*	-20.5 (-53.6, 2.6)*

CI: confidence interval, UACR: urinary albumin-to-creatinine ratio

The values represent the mean (inter-quartile range).

* p < 0.05 vs. baseline

No significant differences were found between the treatment arms.

**Table 4:** Percentage change in the urinary angiotensinogen-to -creatinine ratio.

	DRI		ARB	
%Change in UATGCR (95% CI)	12 weeks	24 weeks	12 weeks	24 weeks
Total	-0.1 (-8.9, 3.8)	-1.7 (-10.4, 7.9)	-5.8 (-17.7, 2.4)*	-5.4 (-22.1, 1.6)*
High-normal albuminuria	-1.1 (-9.1, -1.1)	-2.9 (-13.4, 0.6)*	-5.6 (-14.2, 0.1)*	-3.0 (-18.9, 0.4)*
Microalbuminuria	0.8 (-8.5, 13.7)	1.0 (-3.2, 24.2)	-6.8(-20.5, 8.9)	-8.1 (-28.3, 8.2)

CI: confidence interval, UATGCR: urinary angiotensinogen-to-creatinine ratio

The values represent the mean (inter-quartile range).

* p < 0.05 vs. baseline

No significant differences were found between the treatment arms.

Adverse events were reported in 11 cases (5 in DRI, 6 in ARB). The most commonly reported adverse events were infectious disease, elevated levels of liver enzymes, and gastrointestinal symptoms. Hyperkalemia and elevated serum creatinine levels were not reported.

## Discussion

The relief of microalbuminuria with the blockade of the renin-angiotensin-aldosterone system is known to be a key therapeutic strategy for reducing the risk of renal and cardiovascular events in patients with diabetes. In addition, even within the normal range (< 30 mg/g), ACR ≥ 10 mg/g was associated with a significantly greater rate of decline in renal function than ACR < 10 [12]. In the present study, both DRI and ARB showed significant reductions in the UACR in patients with type 2 diabetes and HNA or MA. The degree of reduction in the UACR was not markedly different between two treatment arms. However, in a subgroup analysis, significant reductions in the UACR were found in the ARB arm but not in the DRI arm. These results indicated that DRI was not superior to ARB in the reduction of renal and/or cardiovascular risk in type 2 diabetes patients with HNA or micro MA.

We defined the change in the UATGCR as the secondary outcome. Angiotensinogen is a 52- to 64-kD peptide molecule with limited glomerular permeability [19]. Most of the angiotensinogen is formed in tubule cells and then is secreted into the tubular fluid [4][20]. Because of these properties, urinary AGT has been studied and reported as a marker for intrarenal RAS activity in animal models and human diseases [21]. In addition, it has been demonstrated that the urinary AGT level is associated with an increased risk for the deterioration of renal function in CKD patients [22]. In this study, the UATGCR was significantly reduced in the ARB arm not but in the DRI arm, although the BP control level was similar between both arms. Since increased RAS activity has been reported to play an important role in the hemodynamic and non-hemodynamic mechanisms involved in kidney injury, ARB, but not DRI, may be able to prevent the progression of nephropathy in a blood pressure-independent manner.

In this study, we analyzed the difference in the changes in the ACR and UATGR using an ANOVA model. In addition, we re-analyzed the data using a Generalized Estimating Equations (GEE) model and found that the outcome was not markedly different from that obtained using the ANOVA model.

The major limitation of this study was that the number of patients (n = 247) was smaller than we initially estimated (n = 280). We estimated that, if the mean difference in the reduction in the ACR in the DRI and ARB groups is 20% (standard deviation was estimated to be 55% from our pilot study), we would need to study 120 experimental subjects and 120 control subjects to be able to reject the null hypothesis that the population means of the DRI and ARB groups are equal with 80% power using a *t*-test. Furthermore, assuming that 15% of patients might withdraw or be lost to follow-up, a total of 140 patients per group would be required. However, in this study, we analyzed only 111 subjects in the DRB group and 114 in the ARB group, and our study had 80% power with 5% significance (2-sided) to detect a 21% difference (79% power to detect a 20% difference) in the means of the DRI and ARB groups.

In conclusion, DRI and ARB reduced albuminuria in hypertensive patients with type 2 diabetes. In addition, ARB, but not DRI, reduced albuminuria even in patients with the normo-albuminuric range. DRI is not superior to ARB in the reduction of urinary excretion of albumin and angiotensinogen.

## Supporting Information

S1 Checklist CONSORT checklist of this study. (DOC)

S1 Protocol Study protocol in English. (DOCX)

S2 Protocol Study protocol. (DOCX)
