# Supplementary material for: Comparative Effects of Direct Renin Inhibitor and Angiotensin Receptor Blocker on Albuminuria in Hypertensive Patients with Type 2 Diabetes. A Randomized Controlled Trial
Source: PLoS One. 2016 Dec 29;11(12):e0164936. doi: 10.1371/journal.pone.0164936 (PMC5198982; doi:10.1371/journal.pone.0164936)
Supplement: S1 Protocol — (DOCX) [file pone.0164936.s002.docx]

**Trial for the reduction of microalbuminuria in hyperetensive type 2 diabetes**

**Study Protocol**

***S*higa *M*icro*A*lbuminuria *R*eduction *T*rial-2 (SMART2)**

The Shiga Committee for Preventing Diabetic Nephropathy

# Revision No.

| 1 | 2011/01/25 |
| --- | --- |
| 2 | 2011/02/12 |
| 3 | 2011/02/22 |
| 4 | 2011/04/08 |
| 5 | 2011/05/09 |
| 6 | 2011/07/19 |
| 7 |  |

**Ⅰ．Summary**

**Ⅰ.1. Study Title**

Trial for the reduction of microalbuminuria in hyperetensive type 2 diabetes

**Ⅰ.2. Aim**

To compare the effect of angiotensin receptor blockers (ARB) and direct renin inhibitor (DRI) on the microalbuminuria in patients with type 2 diabetes and hypertension.

**Ⅰ.3. Design**

Randomized, open label, prospective study

**Ⅰ.4. Participants**

Type 2 diabetes and hypertension (treated with antihypertensive agents or sitting BP >130/80nmmHg) and type 2 diabetes (with normo- or micro-albuminuria)

**Inclusion criteria**

Genders: Both

Ages: 20 Years to 75 Years

Outpatients

Hypertension ：Treated with antihypertensive agents or sitting BP >130/80nmmHg

Type 2 diabetes

Urinary albumin excretion：more than 10 mg/g Cr、less than 300 mg/g Cr

Informed consent: Patients who understand well about this study based on own voluntary will and can give a written consent

**Exclusion criteria**

- Patients with severe hypertension (a systolic or diastolic BP of greater than 180 mmHg and 110 mmHg, respectively), malignant hypertension, and known secondary hypertension.
- Patients with type 1 diabetes
- Patients whose investigator regards as difficult to comply with study protocol in reference to the package insert of aliskiren with a history of gastrointestinal tract surgery
- Patients with hyperkalemia at baseline (> 5.6 mEq/L)
- Urinary albumin excretion：< 10 mg/g Cr or >300 mg/g Cr
- Patients who participated in another clinical study within three months

**Ⅰ.5. Methods**

**Antihypertensive therapy**

Patients with treated with RAS inhibitors at beginning of the observation period. (-8 weeks), RAS inhibitors are replaced by other antihypertensive drugs during the observation period. Patients will be randomly assigned to receive either DRI or ARB (any ARB) according to a minimization method.

The target blood pressure (BP) is <130/80 mmHg. If the target BP is not achieved with the initial dosage of study drugs at four weeks of the intervention period, doses will be titrated up the drugs. (Figure 1). If necessary, additional antihypertensive drugs, other than RAS inhibitors and potassium-retaining diuretics, can be added

Patients were followed up every 4 weeks for 24 weeks, and blood and urine samples were collected at 12 and 24 weeks.

**Ⅰ.6. Outcome mesures**

Primary Outcome Measures:

Reduction in albuminuria:

Change in the urinary albumin to creatinine ratio (UACR) from the baseline

Secondary Outcome Measures:

Change in the urinary angiotensinogen level

Change in the plasma renin activity

Change in the serum insulin level

Following parameters will be measured by the central laboratories.

Urinary albumin, serum and urinary angiotensinogen, urinary creatinine, plasma renin activity,

HbA1c, fasting blood sugar, fasting plasma insulin

Adverse events, medication, and patient compliance are checked and recorded at each visit of the intervention period. Figure 1

**Ⅲ.8. Sample size**

The target sample size for the present study was set at 320 patients (completed study 280 patients) will be needed to give 90% power at a two-sided 5% significance to detect a clinical significant between-groups difference in the primary endpoint.

160 cases　(Ualb/ Ucr 10 mg/gCr<、<30 mg/gCr

160 cases (Ualb/Ucr 30 mg/gCr<、<300 mg/gCr)

**Ⅲ.9. Duration of the study**

August 2011～　March 2013

**Ⅲ.16.1.**

Study Chair: Hiroshi Maegawa （Shiga University of Medical Science）

　　 Chief of Secretariat：Takashi Uzu　（Shiga University of Medical Science）

**Ⅲ.16.4.** Data monitoring: Mayumi Yamanaka

**Ⅲ.17.** This study will be registered with ClinicalTrials.gov
