# Supplementary material for: Comparative Effects of Direct Renin Inhibitor and Angiotensin Receptor Blocker on Albuminuria in Hypertensive Patients with Type 2 Diabetes. A Randomized Controlled Trial
Source: PLoS One. 2016 Dec 29;11(12):e0164936. doi: 10.1371/journal.pone.0164936 (PMC5198982; doi:10.1371/journal.pone.0164936)
Supplement: S2 Protocol — (DOCX) [file pone.0164936.s003.docx]

**2型糖尿病を合併する高血圧患者における**

**微量アルブミン尿抑制効果に関する臨床研究**

**－Stage2－**

**研究実施計画書**

***S*higa *M*icro*A*lbuminuria *R*eduction *T*rial-2 (SMART2)**

**滋賀糖尿病性腎症進展予防研究会**

**SMART2運営委員会事務局**

# Revision No.

| 1 | 2011/01/25 |
| --- | --- |
| 2 | 2011/02/12 |
| 3 | 2011/02/22 |
| 4 | 2011/04/08 |
| 5 | 2011/05/09 |
| 6 | 2011/07/19 |
| 7 |  |

目　次

Ⅰ．研究計画の要約　．．．．．．．．．．．．．．．．．．．．．．．．．．．．．．．．．．．．．．．．．．．．．．P.3

Ⅱ．研究計画の経緯　．．．．．．．．．．．．．．．．．．．．．．．．．．．．．．．．．．．．．．．．．．．．．．P.6

Ⅲ．研究計画　．．．．．．．．．．．．．．．．．．．．．．．．．．．．．．．．．．．．．．．．．．．．．．．．．．．．P.7

　1．研究の目的　．．．．．．．．．．．．．．．．．．．．．．．．．．．．．．．．．．．．．．．．．．．．．．．．．P.7

　2．研究デザイン　．．．．．．．．．．．．．．．．．．．．．．．．．．．．．．．．．．．．．．．．．．．．．．．P.7

3．研究対象　．．．．．．．．．．．．．．．．．．．．．．．．．．．．．．．．．．．．．．．．．．．．．．．．．．．P.7

3.1.対象　．．．．．．．．．．．．．．．．．．．．．．．．．．．．．．．．．．．．．．．．．．．．．．．．．．．． P.7

3.2.選択基準　．．．．．．．．．．．．．．．．．．．．．．．．．．．．．．．．．．．．．．．．．．．．．．．． P.7

3.3.除外基準　．．．．．．．．．．．．．．．．．．．．．．．．．．．．．．．．．．．．．．．．．．．．．．．． P.7

3.4.中止・脱落基準　．．．．．．．．．．．．．．．．．．．．．．．．．．．．．．．．．．．．．．．．．． P.8

　4．研究方法　．．．．．．．．．．．．．．．．．．．．．．．．．．．．．．．．．．．．．．．．．．．．．．．．．．．P.8

　　4.1.被験薬．．．．．．．．．．．．．．．．．．．．．．．．．．．．．．．．．．．．．．．．．．．．．．．．．．．． P.8

　　4.2.用法・用量．．．．．．．．．．．．．．．．．．．．．．．．．．．．．．．．．．．．．．．．．．．．．．．．． P.8

　　4.3.併用療法．．．．．．．．．．．．．．．．．．．．．．．．．．．．．．．．．．．．．．．．．．．．．．．．．． P.8

5．研究スケジュール及び観察・検査項目．．．．．．．．．．．．．．．．．．．．．．．．．．． P.9

　5.1.研究スケジュール．．．．．．．．．．．．．．．．．．．．．．．．．．．．．．．．．．．．．．．．．． P.9

　5.2.観察・検査項目．．．．．．．．．．．．．．．．．．．．．．．．．．．．．．．．．．．．．．．．．．．．．P.10

　6．評価　．．．．．．．．．．．．．．．．．．．．．．．．．．．．．．．．．．．．．．．．．．．．．．．．．．．．．．P.12

6.1.有効性の評価．．．．．．．．．．．．．．．．．．．．．．．．．．．．．．．．．．．．．．．．．．．．． P.12

　　6.2.安全性の評価．．．．．．．．．．．．．．．．．．．．．．．．．．．．．．．．．．．．．．．．．．．．． P.12

7．安全性に関わる処置．．．．．．．．．．．．．．．．．．．．．．．．．．．．．．．．．．．．．．．．．．P.12

7.1.有害事象が発現した場合の処置　．．．．．．．．．．．．．．．．．．．．．．．．．．． P.12

7.2.中止時の処置．．．．．．．．．．．．．．．．．．．．．．．．．．．．．．．．．．．．．．．．．．．．． P.12

8．目標症例数　．．．．．．．．．．．．．．．．．．．．．．．．．．．．．．．．．．．．．．．．．．．．．．．． P.13

9．研究実施期間　．．．．．．．．．．．．．．．．．．．．．．．．．．．．．．．．．．．．．．．．．．．．．． P.13

10. 統計解析．．．．．．．．．．．．．．．．．．．．．．．．．．．．．．．．．．．．．．．．．．．．．．．．．．．． P.13

11．研究管理事項．．．．．．．．．．．．．．．．．．．．．．．．．．．．．．．．．．．．．．．．．．．．．．．． P.13

11.1.研究計画書の不遵守（逸脱または変更等）発生時の処置　．．．．． P.13

11.2.研究計画書の変更等に関する手順　．．．．．．．．．．．．．．．．．．．．．．．．． P.13

11.3.金銭の支払い及び健康被害補償 ．．．．．．．．．．．．．．．．．．．．．．．．．．．． P.13

11.4.研究の中止、中断または終了．．．．．．．．．．．．．．．．．．．．．．．．．．．．．．．．P.14

12.倫理．．．．．．．．．．．．．．．．．．．．．．．．．．．．．．．．．．．．．．．．．．．．．．．．．．．．．．．．．．P.14

　12.1.倫理委員会．．．．．．．．．．．．．．．．．．．．．．．．．．．．．．．．．．．．．．．．．．．．．．．． P.14

　　12.2.患者の保護．．．．．．．．．．．．．．．．．．．．．．．．．．．．．．．．．．．．．．．．．．．．．．．． P.14

　　12.3.個人情報を含む情報の保護についての具体的方法．．．．．．．．．．．．．． P.14

　　12.4.被験者からの情報の開示請求　．．．．．．．．．．．．．．．．．．．．．．．．．．．．．． P.14

　13.インフォームドコンセント．．．．．．．．．．．．．．．．．．．．．．．．．．．．．．．．．．．．． P.15

　　13.1.被験者への説明．．．．．．．．．．．．．．．．．．．．．．．．．．．．．．．．．．．．．．．．．．．． P.15

　　13.2.被験者の同意．．．．．．．．．．．．．．．．．．．．．．．．．．．．．．．．．．．．．．．．．．．．．．．P.15

　14.プロトコールの遵守．．．．．．．．．．．．．．．．．．．．．．．．．．．．．．．．．．．．．．．．．．． P.15

　15.研究成果の発表．．．．．．．．．．．．．．．．．．．．．．．．．．．．．．．．．．．．．．．．．．．．．．． P.15

　16.研究組織．．．．．．．．．．．．．．．．．．．．．．．．．．．．．．．．．．．．．．．．．．．．．．．．．．．．． P.15

　　16.1.研究総括医師／事務局代表．．．．．．．．．．．．．．．．．．．．．．．．．．．．．．．．．． P.16

　　16.2.研究参加施設／研究責任医師．．．．．．．．．．．．．．．．．．．．．．．．．．．．．．．． P.16

　　16.3.効果安全性勧告委員会（DSMB）．．．．．．．．．．．．．．．．．．．．．．．．．．．．． P.16

　　16.4.データマネジメント．．．．．．．．．．．．．．．．．．．．．．．．．．．．．．．．．．．．．．．． P.16

　17.国際登録．．．．．．．．．．．．．．．．．．．．．．．．．．．．．．．．．．．．．．．．．．．．．．．．．．．．． P.16

　18.資金源．．．．．．．．．．．．．．．．．．．．．．．．．．．．．．．．．．．．．．．．．．．．．．．．．．．．．．． P.16

　19.利益相反．．．．．．．．．．．．．．．．．．．．．．．．．．．．．．．．．．．．．．．．．．．．．．．．．．．．． P.16

**Ⅰ．研究計画の要約**

**Ⅰ.1. 課題名**

2型糖尿病を合併する高血圧患者における微量アルブミン尿抑制効果に関する臨床研究 - SMART2 Stage2

**Ⅰ.2. 研究の目的**

2型糖尿病を合併する高血圧患者に対し、2種のRAS系阻害薬「アンジオテンシンⅡ受容体拮抗薬；ARB」と「直接的レニン阻害薬；DRI（アリスキレン）」により厳格な血圧管理下にて尿中アルブミン量抑制効果を比較検討する。

**Ⅰ.3. 研究デザイン**

前向き非遮蔽無作為化並行群間比較試験

**Ⅰ.4. 対象**

2型糖尿病を合併する高血圧（降圧薬治療中あるいは安静座位による平均の収縮期血圧130 mmHg and/or 拡張期血圧 80 mmHg以上）患者のうち、糖尿病性腎症Ⅰ期、Ⅱ期の患者（糖尿病性腎症早期診断基準　厚生省平成2年度糖尿病調査研究報告書248-251）

**Ⅰ.5. 研究方法**

１）降圧療法

観察期開始時（-8週）にRAS系阻害薬を服用している患者は、原則的に、RAS系阻害薬以外の降圧薬治療に切替え、治療期開始時（0週）に適格と判定された場合、アリスキレン群、ARB群のいずれかに無作為に割付け^＊^られ、アリスキレン150 mg/dayあるいは各種ARBの常用量にて治療を開始する。

治療期開始前8週間以内に降圧薬の服用がなく、治療期開始時（0週）に適格と判定された患者は、アリスキレン群、ARB群のいずれかに無作為に割付け^＊^られ、アリスキレン150 mg/dayあるいは各種ARBの常用量にて治療を開始する。

治療期開始以降、原則4週毎に血圧を測定し目標血圧（SBP/DBP：130/80 mmHg未満）に到達しない場合、アリスキレンまたは各種ARBを増量する。増量によっても目標血圧値に到達しない場合、RAS系阻害薬（DRI、ARB、ACE-I）及びカリウム保持性利尿薬以外の降圧薬を最小用量から併用する。過度の降圧を避けるため降圧薬の用量は減量中止の処置を含めフレキシブルに行うものとする。

^＊^無作為割り付け： 観察期（-4週）に測定した尿中アルブミン量及び被験者背

　　　　　　　　　　　 景によって、最小化法を用いて無作為にアリスキレン群、ARB

　　　　　　　　　　　 群のいずれかに割り付ける。

２）研究期間

観察期：治療開始時（0週）までの期間を「観察期」とし8週間観察を行う。この間に試験への適格性を確認するとともに必要な検査を実施する。

治療期：治療開始後の期間を「治療期」とし24週間観察する。この間に必要な検査、有害事象の調査など行う。

　　原則的に、観察期から治療期を通じて診察時刻は一定とし、空腹かつ治療薬を服薬せず外来診察を行う。（来院日は診察後服薬を原則とする）

**Ⅰ.6. 研究スケジュールと評価項目**

１）有効性評価項目

〔主要評価項目〕

尿中アルブミン量の変化率

尿中アルブミン量の正常化率及び50%減少率。

〔副次的評価項目〕

尿中アンジオテンシノージェン、血中アンジオテンシノージェン、

血漿レニン活性、血中インスリンの変化

臨床検査：各施設で測定する（保険診療範囲）。

血液学検査、生化学検査

指定特殊検査：研究会が指定する中央検査センターにて測定する。

尿中アルブミン、尿中アンジオテンシノージェン、尿中クレアチニン、血中ア

ンジオテンシノージェン、血漿レニン活性、HbA1c、空腹時血糖、空腹時血中

インスリン

２）安全性評価項目

有害事象（自覚症状、他覚所見の新たな発現または悪化、臨床検査値の異常変動）

３）研究スケジュール

**Ⅰ.7. 目標症例数**

計320例（観察完了例280例）

尿中アルブミン量が10 mg/gCr以上300 mg/gCr未満の患者

〔糖尿病性腎症^※^〕

Ⅰ期　160例　(尿中アルブミン量10 mg/gCr以上、30 mg/gCr未満）

Ⅱ期　160例　(尿中アルブミン量30 mg/gCr以上、300 mg/gCr未満)

※I期、Ⅱ期の判定は、観察期-4週の尿中アルブミン値を用いて治療期開始時（0週）に判定する。いずれの群も目標症例数に達した時点で登録終了とする。

**Ⅰ.8. 研究実施期間**

2011年8月　～　2013年3月

- 本研究は、糖尿病性腎症進展予防研究会（代表世話人：柏木厚典、事務局：滋賀医科大学　糖尿病代謝・腎臓・神経内科）主導による、「研究者主導型研究」である。

運営委員会　研究総括医師

前川　聡　　（滋賀医科大学　糖尿病代謝・腎臓・神経内科）

運営委員会　事務局代表

宇津　貴　　（滋賀医科大学　糖尿病代謝・腎臓・神経内科）

**Ⅱ．研究計画の経緯**

　糖尿病、高血圧はともに心血管疾患のリスクファクターであり、両者は高頻度に合併することがよく知られている。また、慢性腎臓病（CKD）に移行する患者が多いことも知られている。このような合併患者に対しては、血糖コントロールのみならず、厳格な血圧コントロールが重要であるとされ、米国糖尿病ガイドライン（ADA；2001年）、高血圧ガイドライン（JNC7；2003年）によって推奨されている。同様に、CKD（慢性腎臓病）治療ガイドにおいて、血圧のコントロールはCKDの基本の治療となっている。糖尿病患者、CKD患者における降圧目標値は、収縮期130 mmHg未満かつ拡張期80 mmHg未満とされており、降圧薬は原則としてRAS系阻害薬（ACE-IかARB）による治療が推奨されている。

一方わが国における糖尿病罹患率は年毎に上昇しており、続発する透析導入率上昇は医療行政や患者のQOLに与える影響も大きい。以上のことから糖尿病性腎症のⅡ期までの治療に成功すれば将来的透析導入率を低下させる可能性があると考えられる。

我々は2007年2型糖尿病を伴う高血圧患者に対しShiga Microalbuminuria Reduction Trial（SMART試験）の結果を公表し、アンジオテンシンⅡ受容体拮抗薬（ARB）Valsartanによる降圧療法がCa拮抗薬Amlodipinに比し有意に微量アルブミン尿を有意に減少させることを公表した。

　2009年、本邦においてもレニン阻害薬（DRI）Aliskirenが医療の場で使用できることになった。DRIはRASの起点に位置する律速酵素を直接的に阻害することによりAng1以降のアンジオテンシンペプチドの産生を強力に抑制する新しい作用機序の降圧剤である。国内外で多くの臨床試験が行われ、血圧コントロールに優れ臓器保護作用があることが示唆されている。海外で行われたALOFT、AVOIDの試験結果から、微量アルブミン尿を減少させることが期待されている。降圧薬の腎保護に関する多くの臨床研究では、糖尿病性腎症Ⅱ期を対象とした研究がほとんどでありⅠ期の症例での腎保護効果がもつ意義が検討される必要がある。近年、尿中、血中アンジオテンシノージェンが測定可能となり、治療効果に対するメカニズム解明の手段が増え、より詳細な検討が可能となった。

以上のことから、我々は、少数例でアリスキレン用い腎保護効果を検討し（SMART-2研究Stage1）、これら成績を基にSMART-2研究Stage2の研究デザインを策定し、臨床検討を行うこととした。

**Ⅲ．研究計画**

**Ⅲ.1. 研究の目的**

2型糖尿病を合併する高血圧患者に対し、2種のRAS系降圧薬（アンジオテンシンⅡ受容体拮抗薬（ARB）と直接的レニン阻害薬（DRI）Aliskiren）により厳格な血圧管理下にて尿中アルブミン量抑制効果を比較検討する。

**Ⅲ.2. 研究デザイン**

前向き非遮蔽無作為化並行群間比較試験

**Ⅲ.3. 研究対象**

**Ⅲ.3.1. 対象**

2型糖尿病を合併する高血圧（降圧薬治療実施中あるいは安静座位による平均の収縮期血圧130 mmHg and/or 拡張期血圧80 mmHg以上）患者のうち糖尿病性腎症Ⅰ期、Ⅱ期の患者

（糖尿病性腎症早期診断基準　厚生省平成2年度糖尿病調査研究報告書248-251）．

**Ⅲ.3.2. 選択基準（以下のすべてを満足する患者）**

性別　 ：制限なし

年齢　 ：20歳以上75歳未満

区分　 ：外来受診患者

高血圧 ：降圧薬にて治療中、あるいは収縮期血圧130 mmHg and/or

拡張期血圧80 mmHg以上の患者

2型糖尿病 ：ADA（米国糖尿病学会）基準で2型糖尿病と診断される患者もしくは糖尿病治療中の患者

尿中アルブミン量：10 mg/g･Cr以上、300 mg/g･Cr未満の患者

インフォームドコンセント：本人の自由意思による研究への充分な理解があり、文書による同意取得が可能な患者

**Ⅲ.3.3. 除外基準（以下の患者は対象から除外する）**

- 重症高血圧患者（180/110 mmHg以上）、悪性高血圧患者、二次性高血圧患者
- 1型糖尿病患者
- アリスキレンの「使用上の注意」から、担当医師が本研究実施計画書を遵守するのが困難であると判断した患者
- 薬剤の吸収を阻害するような胃腸系の手術、胃腸障害の既往、合併を有する患者
- 高カリウム（5.6 mEq/L以上）を呈する患者
- 尿中アルブミン量10 mg/g･Cr未満、300 mg/g･Cr以上の患者
- 観察期開始前3ヶ月以内に治験に参加した患者
- その他、本研究計画書の遵守が困難である患者

**Ⅲ.3.4. 中止・脱落基準**

中止　担当医師の判断により医学的に継続が困難とされた場合、観察を中止し、

　　　理由を記録する。

脱落　患者本人の意思または事由により試験から離脱する場合、脱落と扱い、理由を記録する。

**Ⅲ.4. 研究方法**

**Ⅲ.4.1. 被験薬・対照薬**

被験薬　アリスキレン　150 mg錠

対照薬　国内で販売されている各種ARB

**Ⅲ.4.2. 用法・用量**

観察期開始時（-8週）にアリスキレンまたはARBを服薬している患者は、原則的に、RAS系阻害薬以外の降圧薬治療に切替え、治療期開始時（0週）に適格と判定された場合、アリスキレン群、ARB群のいずれかに無作為に割付け*られ、アリスキレン150 mg/dayあるいは各種ARBの常用量にて治療を開始する。

治療期開始前8週間以内に降圧薬の服薬がなく、治療期開始時（0週）に適格と判定された患者は、アリスキレン群、ARB群のいずれかに無作為に割付け*られ，アリスキレン150 mg/dayあるいは各種ARBの常用量にて治療を開始する。

治療期開始以降、原則4週毎に血圧を測定し目標血圧（SBP/DBP：130/80 mmHg未満）に到達しない場合、アリスキレンまたは各種ARBを増量する。増量によっても目標血圧値に到達しない場合、RAS系阻害薬（DRI、ARB、ACE-I）及びカリウム保持性利尿薬以外の降圧薬を最小用量から併用する。過度の降圧を避けるため降圧薬の用量は減量中止の処置を含めフレキシブルに行うものとする。

　注意点：アリスキレン服用時刻は食後服用するよう指導することが望ましい。

^＊^無作為割り付け： 観察期（-4週）に測定した尿中アルブミン量及び被験者背

　　　　　　　　　　　 景によって、最小化法を用いて無作為にアリスキレン群、ARB

　　　　　　　　　　　 群のいずれかに割り付けられる。

**Ⅲ.4.3. 併用療法**

既存治療薬　：観察期開始前までに使用した降圧薬、基礎疾患治療薬のうち、臨床評価に影響を及ぼすと思われる薬剤の種類、用量を記録する。

服薬禁止薬　：治療期開始前8週間以内に次の降圧薬を服薬中の患者は、治療期へ移行できない。

　　　　 RAS系阻害薬（DRI、ARB、ACE-I）

治療期期間中、両群で次の降圧薬の併用は禁止とする。

RAS系阻害薬（DRI、ARB、ACE-I）、カリウム保持性利尿薬

併用可能薬： アリスキレンまたは各種ARBの1日最大用量にて目標血圧値に到達できない場合には、RAS系阻害薬（DRI、ARB、ACE-I）及びカリウム保持性利尿薬以外の降圧薬を併用可とする。また観察期より合併症治療の目的で使用されている薬剤はその用法用量を変えずに併用することを可能とする。

**Ⅲ.5. 研究スケジュール及び観察・検査項目**

**Ⅲ.5.1. 研究スケジュール**

以下の研究スケジュールに従って観察・検査を行う。

観察期：治療開始までの期間を「観察期」とし8週間観察を行う。この間に試

験への適格性を確認するとともに必要な検査を実施する。

治療期：治療開始後の期間を「治療期」とし24週間観察する。この間に必要

な検査、有害事象の調査等を行う。

　観察期から治療期を通じて検査を伴う外来では、可能な限り診察時刻は一定とし、空腹かつ服薬せず外来診察を行う。（外来日の服薬は診察後を原則とする）

**Ⅲ.5.1.1. 同意取得**

　本研究へ参加される被験者に、観察開始前に同意説明文書を用い本研究の内容を十分に説明し被験者本人の自由意思による同意文書を取得する。

**Ⅲ.5.1.2. 被験者背景因子の調査**

　年齢、性別、身長、体重、文書同意取得日、診断名、合併症、既往歴、喫煙を

含む生活習慣諸因子、既治療薬（薬剤種類、投与量）について調査・記録する。

**Ⅲ.5.1.3. 選択基準の確認**

　治療開始前に、被験者の研究対象条件を最終確認し選択基準条件を満たしていない場合は、治療期へ移行できない。

**Ⅲ.5.2. 観察・検査項目**

**Ⅲ.5.2.1. 座位血圧／脈拍数**

来院時に、座位血圧及び脈拍数を測定する。

〔座位血圧測定方法〕

原則として、座位にて5分間以上安静を保った後、座位血圧を1-2分間隔で3回測定し、後半2回の測定値の平均を記録する。（同一被験者に対し、測定者及び計測条件を変更しないことが望ましい）

〔脈拍測定方法〕

座位血圧測定直前に1回実施する。測定時間は30秒とし、1分間の換算値を記録する。

**Ⅲ.5.2.2. 身長・体重**

　治療期開始時（0週）に身長・体重を計測・記録する。

**Ⅲ.5.2.3. 尿中アルブミン量（mg/g・Cr）**

　治療期開始4週前（-4週）及び治療期間（12週、24週）の各来院時に、尿中アルブミン量（早朝第一尿）を測定する。なお、評価のためにそれぞれ3回（来院当日及び前日、前々日に採尿）測定する。

-4週の測定値が10 mg/g・Cr以上、300 mg/g・Cr未満であることを確認することによって治療期へ移行できる。

-8週、8週、20週の来院時に、被験者に採尿容器を手渡し、次回の来院日に3日分の早朝第一尿を採取し、持参するよう依頼する。なお、採尿サンプルを回収した後は、速やかに指定の臨床検査会社に連絡して当該サンプルの回収、測定を依頼する。

**Ⅲ.5.2.4. 臨床検査**

　治療期開始時（0週または-4週）、12週、24週（終了時）に血液を採取し、血液学・生化学検査を実施する。検査は空腹にて実施する。

＊K値の変動には留意し観察期に比べ顕著に上昇していた場合には適切な処置を行うこと。

〔検査項目〕

血液学検査： 白血球数、赤血球数、Hb、Ht、血小板数

生化学検査： 総蛋白、総ビリルビン、BUN、血清クレアチニン、尿酸、AST（GOT）、ALT(GPT)、γ-GTP、LDH、ALP、総コレステロール、TG、HDL、Na、K、Cl、Ca、P、

　＊各施設で測定する（保険診療範囲）。

**Ⅲ.5.2.5. 指定特殊検査**

　観察期-4週、及び治療期12週、24週（終了時）に検査を施行する。採尿、採血サンプルを回収した後は、速やかに検査会社に連絡して当該サンプルの回収、測定を依頼する。

〔検査項目〕

尿中アルブミン、尿中アンジオテンシノージェン、尿中クレアチニン、血中アン

ジオテンシノージェン、血漿レニン活性、HbA1c、空腹時血糖、空腹時血中イン

スリン

　＊研究会が指定する中央検査センターにて測定する。

**Ⅲ.5.2.6. 有害事象**

　治療期間中の有害事象の発現有無を確認する。有害事象が発現した場合は、速やかに適切な処置・治療を施すとともに内容詳細を調査し、記録する。

　 また、グレード3以上の有害事象が認められた場合には、速やかに研究事務局に

連絡の上、安全性委員会及び倫理委員会に報告する。

**Ⅲ.5.2.7. 併用治療**

治療期間中に、新たな併用治療の開始、併用治療の変更等が生じた場合は、その内容と理由を記録する。

**Ⅲ.5.2.8. 服薬状況の確認**

各来院時に降圧薬の服薬状況を確認する。

**Ⅲ.6. 評価**

**Ⅲ.6.1. 有効性の評価**

〔主要評価項目〕 尿中アルブミン量の変化率

尿中アルブミン量の正常化率及び50%減少率

〔副次的評価項目〕　 尿中アンジオテンシノーゲン、血漿レニン活性及び血中インスリンの変化

**Ⅲ.6.2. 安全性の評価**

　有害事象（自覚症状、他覚所見の新たな発現または悪化、臨床検査値の異常変動）について記録し検討する。

〔有害事象と副作用〕

　有害事象とは、研究期間中に発現した健康上好ましくない事象すべてであり、被験薬との因果関係は問わない。有害事象のうち、被験薬との因果関係が否定されないものは副作用として取り扱う。

**Ⅲ.7. 安全性に関わる処置**

**Ⅲ.7.1. 有害事象が発現した場合の処置**

　 有害事象が発生した場合は、中止を含めた適切な処置・治療を行うとともに速やかに有害事象報告を行う（事象名、程度、重篤度、発現日、転帰、被験薬との因果関係、処置を調査・判定し、記録する）。発現した有害事象は、可能な限りその有害事象が回復するまで、あるいは更なる経過観察が不要と判断できるまで経過観察する。また、中止理由、処置、その他の経過等を可能な限り追跡調査する。

「医薬品に関わる有害事象」は、当該薬を製造販売する各製薬会社に連絡する。有害事象によって観察の継続が困難と判断される場合には医師の判断により観察を中止する。

重篤な有害事象が発現し、研究総括医師が経過観察の中止を判断した場合には、速やかにその旨を各医療機関の長に報告する。

**Ⅲ. 7.2. 中止時の処置**

臨床研究により期待される利益よりも危険度が高いと医師が判断する場合には、本研究における治療を中止する。有害事象の発現により中止した場合は、「Ⅲ.7.1.有害事象が発現した場合の処置」に従い適切な処置を行うこと。また、中止理由等詳細を記録する。

**Ⅲ.8. 目標症例数**

320例（観察完了例280例）

DRIがARBに優れる（優越性）仮説を検証するための必要例数を尿中アルブミン

量が300 mg/gCr未満の患者から得ることとし、算出した目標患者数を推定した。

〔糖尿病性腎症^※^〕

Ⅰ期　160例　(尿中アルブミン量10 mg/gCr以上、30 mg/gCr未満）

Ⅱ期　160例　(尿中アルブミン量30 mg/gCr以上、300 mg/gCr未満)

※I期、Ⅱ期の判定は、観察期-4週の尿中アルブミン値を用いて治療期開始時（0週）に判定する。いずれの群も目標症例数に達した時点で登録終了とする。

**Ⅲ.9. 研究実施期間**

2011年8月　～　2013年3月

**Ⅲ.10. 統計解析**

　試験とは独立した解析機関にて解析する。

**Ⅲ.11. 研究管理事項**

**Ⅲ.11.1. 研究計画書の遵守(逸脱または変更等)発生時の処置**

担当医師は、原則としてIRBの事前承認なしに研究計画書からの逸脱または変更を行ってはならない。但し被験者の危険を回避するため等やむをえない場合はその限りではないが、速やかに逸脱・変更の理由を研究事務局及び医療機関の長に提出する。

**Ⅲ.11.2. 研究計画書の変更等に関する手順**

研究計画書の変更の必要性が生じた場合は、事務局で協議し決定する。変更点についてはIRBへ報告する

**Ⅲ.11.3. 金銭の支払い及び健康被害補償**

**Ⅲ.11.3.1. 患者への費用負担**

本研究期間中に発生する診療諸費用は日常診療の範囲内であることから、本研究での保険診療の範囲内での診療に関する費用は患者負担とする。ただし、日常診療範囲外の特殊検査に掛かる費用は試験を実施する研究会が負担する。

**Ⅲ.11.3.2. 健康被害補償・補償保険**

本研究中に被験者に健康被害が生じた場合は、速やかに適切な処置、治療を行う。

また、重篤な副作用等の健康被害が生じた場合は、医薬品副作用被害救済制度を適用し、救済給付の申請を行う。また本研究は「補償保険」に加入する。

**Ⅲ.11.4. 研究の中止、中断または終了**

本研究の途中で研究全体を中止せざるを得ない理由が生じた場合、研究総括医師は中止理由を研究担当医師に連絡する。また、研究責任医師は、その旨及びその理由を速やかに医療機関の長に連絡する。

**Ⅲ.12. 倫理**

**Ⅲ.12.1. 倫理委員会**

本研究は滋賀医科大学の倫理委員会で承認を得て実施する。参加施設は各施設の倫理委員会の承認を得て実施する。実施計画書及び被験者への説明文書を改訂する場合も倫理委員会の承認を必要とする。

**Ⅲ.12.2. 患者の保護**

本研究に関係するすべての研究者はヘルシンキ宣言及び厚生労働省「臨床研究に関する倫理指針」(<http://imcj.go.jp/rinri/index.html>)を遵守して、本研究を実施する。

１）症例の集積及び解析に際して患者個人名は特定できない。

２）本研究が公表される場合も被験者の秘密は保全される。

３）得られたデータは本研究目的以外に使用されない。

**Ⅲ.12.3. 個人情報を含む情報の保護についての具体的方法**

本研究にかかわるものは、参加するすべての患者の個人情報を保護する

　　１）研究資料には住所や氏名など個人を特定できる情報は含まれず、符号を付けて

　　匿名化管理する。匿名化した符号と患者の個人情報とを連結させる対応表は、

　　担当医が厳重に保管する。

　　２）個人を特定できる情報を含まない研究結果、資料や診療記録、個人情報は対応

　　　　表とは別に管理する。

　　３）本研究で使用されるコンピュータは診療用を含む他のコンピュータと切り離さ

　　　　れたものを利用する。

　　４）データは外部記憶装置に保管して独立した鍵のかかる場所で厳重に管理し外部

　　　　への持ち出しは禁止する。

**Ⅲ.12.4. 被験者からの情報の開示請求**

本人が情報の開示を希望する場合は、原則的に結果を開示するが、本人が情報の開示を希望していない場合、本人以外が情報の開示を希望する場合では開示しない。

**Ⅲ.13. インフォームドコンセント**

**Ⅲ.13.1. 被験者への説明**

研究開始に先立ち担当医は施設の倫理委員会承認が得られた同意説明文書を用

いて十分に研究内容についての説明を行う。

**Ⅲ.13.2. 被験者の同意**

1. 研究内容についての説明の後、被験者自身が試験の内容をよく理解したことを

確認し、研究への参加を依頼する。本研究への参加は自由意思にもとづくこと、

参加しない場合でも不利益な対応を受けないこと、参加を表明した後、いつで

も参加を撤回できることなどを、十分に説明する。

1. 被験者が研究参加に同意した場合、所定の同意書を用い、自署されたものを取

得する。

３）同意は，研究参加以前に取得する。

**Ⅲ.14. プロトコールの遵守**

本研究に参加する研究者は、患者の安全と人権を損なわない限りにおいて本プロトコールを遵守する。

**Ⅲ.15. 研究成果の公表**

本研究は学会、論文にて公表を予定しているが、被験者のプライバシーは保全される。また当該学会、投稿雑誌の規定に従い結果の公表に関する守秘義務を遵守する。必要に応じて「守秘義務契約」を締結する。

**Ⅲ.16. 研究組織と運営**

研究組織：滋賀糖尿病性腎症進展予防研究会

研究形態：医師主導型臨床研究（IIT）

　研究実績：2010年度　SMART2研究Stage1を実施

　　　滋賀糖尿病性腎症進展予防研究会－糖尿病性腎症の悪化進展防止に関する臨床研究組織

　　代表世話人：滋賀医科大学病院　院長　　　　　柏木　厚典

　　 運営小委員会：委員長　 滋賀医科大学　前川　聡

委員　 滋賀医科大学　柏木　厚典

委員 滋賀医科大学　宇津　貴

委員 滋賀医科大学　荒木 信一

運営委員会：運営委員長/研究総括医師 滋賀医科大学　前川　聡

運営委員　 　　 　 滋賀医科大学　柏木　厚典

　　　運営委員/事務局代表　　 滋賀医科大学　宇津　貴

　　　　　　　　　　　運営委員/事務局　　　　 滋賀医科大学　荒木 信一

　　　　　　　運営委員　　　　　　　 旭川医科大学　羽田　勝計

　　　　　　　運営委員　　　　　　 金沢医科大学　古家　大祐

　　　　　　　　　　 　※運営委員は運営小委員会の推薦により決定する。

本研究において運営上、組織形成が必要または不要となった場合は運営小委員会で

　検討して決定するものとする。

**Ⅲ.16.1. 研究総括医師／事務局**

研究総括医師：前川　聡 （滋賀医科大学　糖尿病代謝・腎臓・神経内科）

　　 事務局代表：宇津　貴　（滋賀医科大学　糖尿病代謝・腎臓・神経内科）

**Ⅲ.16.2. 研究参加施設/研究責任医師**

　別添「研究参加施設・研究責任医師一覧」参照

**Ⅲ.16.3.　効果安全性勧告委員会（DSMB）**

　　DSMBは本研究全体の有効性及び安全性について監査し、研究の続行または中止勧告を運営委員会に明示する。

**Ⅲ.16.4. データマネージメント**

山中真由美（滋賀医科大学　糖尿病代謝・腎臓・神経内科）

**Ⅲ.17. 国際登録**

　　本研究はClinicalTrials.govに国際登録を行う。

**Ⅲ. 18. 基金源**

　　社団法人　日本糖尿病財団

　　（滋賀医科大学の倫理委員会で承認後、SMART2 Stage2の総合調査研究助成申請を行う）

**Ⅲ.19. 利益相反**

　　成績を公表する学会、投稿する学会の規則に従って検討し必要事項をすべて開示する。
